# Discovery of microvascular miRNAs using public gene expression data: miR-145 is expressed in pericytes and is a regulator of Fli1

**DOI:** 10.1186/gm108

**Published:** 2009-11-16

**Authors:** Erik Larsson, Peder Fredlund Fuchs, Johan Heldin, Irmeli Barkefors, Cecilia Bondjers, Guillem Genové, Christelle Arrondel, Pär Gerwins, Christine Kurschat, Bernhard Schermer, Thomas Benzing, Scott J Harvey, Johan Kreuger, Per Lindahl

**Affiliations:** 1 Wallenberg Laboratory for Cardiovascular Research, Bruna Stråket 16, Sahlgrenska University Hospital, SE-413 45 Gothenburg, Sweden; 2 Institute of Biomedicine, University of Gothenburg, SE-405 30 Gothenburg, Sweden; 3 Department of Medical Biochemistry and Microbiology, Uppsala University, Husargatan 3, SE-751 23 Uppsala, Sweden; 4 Department of Medical Biochemistry and Biophysics, Division of Matrix Biology, Lab of Vascular Biology, Karolinska Institutet, Scheeles väg, 2 A:3-P:4, SE-171 77 Stockholm, Sweden; 5 Inserm U574, Hôpital Necker-Enfants Malades, Equipe Avenir Tour Lavoisier, 6e étage, 149 rue de Sèvres, 75015 Paris, France; 6 Université Paris Descartes, Hôpital Necker-Enfants Malades, Equipe Avenir Tour Lavoisier, 6e étage, 149 rue de Sèvres, 75015 Paris, France; 7 Department of Medicine and Centre for Molecular Medicine, University of Cologne, Kerpener Str. 62, 50937 Köln, Germany; 8 Cologne Excellence Cluster on Cellular Stress Responses in Aging-Associated Diseases, University of Cologne, Kerpener Str. 62, 50937 Köln, Germany

## Abstract

**Background:**

A function for the microRNA (miRNA) pathway in vascular development and angiogenesis has been firmly established. miRNAs with selective expression in the vasculature are attractive as possible targets in miRNA-based therapies. However, little is known about the expression of miRNAs in microvessels *in vivo*. Here, we identified candidate microvascular-selective miRNAs by screening public miRNA expression datasets.

**Methods:**

Bioinformatics predictions of microvascular-selective expression were validated with real-time quantitative reverse transcription PCR on purified microvascular fragments from mouse. Pericyte expression was shown with *in situ *hybridization on tissue sections. Target sites were identified with 3' UTR luciferase assays, and migration was tested in a microfluid chemotaxis chamber.

**Results:**

miR-145, miR-126, miR-24, and miR-23a were selectively expressed in microvascular fragments isolated from a range of tissues. *In situ *hybridization and analysis of *Pdgfb *retention motif mutant mice demonstrated predominant expression of miR-145 in pericytes. We identified the Ets transcription factor Friend leukemia virus integration 1 (Fli1) as a miR-145 target, and showed that elevated levels of miR-145 reduced migration of microvascular cells in response to growth factor gradients *in vitro*.

**Conclusions:**

miR-126, miR-24 and miR-23a are selectively expressed in microvascular endothelial cells *in vivo*, whereas miR-145 is expressed in pericytes. miR-145 targets the hematopoietic transcription factor Fli1 and blocks migration in response to growth factor gradients. Our findings have implications for vascular disease and provide necessary information for future drug design against miRNAs with selective expression in the microvasculature.

## Background

MicroRNAs (miRNAs) are short endogenous RNAs that regulate gene expression through translational repression of specific target mRNA transcripts. miRNAs are transcribed by RNA polymerase II, either from dedicated genes or as parts of introns in host protein coding genes [1]. Maturation begins with trimming of the immediate transcribed product into a stem-loop structure (the pre-miRNA) by the nuclear enzyme Drosha. This is followed by cleavage by the cytosolic enzyme Dicer into a short 19- to 25-bp double-stranded RNA [2]. Normally, one strand is quickly degraded, while the other (the mature miRNA) associates with the RNA-induced silencing complex (RISC). This riboprotein complex has the ability to recognize and silence target mRNAs, usually through imperfect complementarity to sequence elements in the 3' untranslated region (UTR).

Several recent studies establish a role for miRNA in vascular development and angiogenesis [3]. Dicer-deficient mice die during early embryonic development and display impaired angiogenesis and yolk sac formation [4], whereas endothelial-specific inactivation of Dicer reduces postnatal angiogenesis [5]. Small interfering RNA knockdown of Dicer or Drosha leads to reduced endothelial proliferation, sprouting and network formation *in vitro *[6,7]. Moreover, the expression of angiogenesis-related genes, such as *Vegf*, *Flt1*, *Kdr *and *Tie1*, is altered in Dicer mutant embryos [4] and following Dicer knockdown in cultured endothelial cells (ECs) [7]. However, relatively little is known about the function of individual miRNAs in the microvasculature. miR-126 controls VCAM-1 (vascular cell adhesion molecule-1) expression in human umbilical vein endothelial cells (HUVECs) [8] and was recently shown to regulate vascular integrity and angiogenesis *in vivo *[9-11]. Others, including let-7f, miR-27b [6], miR-221, and miR-222 [12], have been shown to modulate angiogenesis *in vitro *and overexpression or inhibition of miR-378 [13], the miR-17-92 cluster [14] and miR-296 [15] affects angiogenesis in mouse engrafted tumors. Some of these studies show direct regulation of a target gene, but downstream mechanisms are in many cases unknown.

In several of the above mentioned studies, microarrays were used to identify mature miRNAs highly expressed in ECs. These experiments were all performed *in vitro *on HUVECs and aimed at the identification of highly expressed miRNAs rather than specific/selective expression [6-8,12], or on embryoid body (EB) cultures [10]. Here, we used publicly available expression datasets to screen for miRNAs with enriched expression in the mature microvasculature *in vivo*. Selected candidates were evaluated using real-time quantitative reverse transcription PCR (qRT-PCR) on mature blood vessel fragments isolated from mouse tissues. miR-145, miR-126, miR-24 and miR-23a were consistently enriched in adult microvessels. We further showed that miR-145 regulated the endothelial Ets factor Fli1 and that miRNA-145 reduced cell migration in response to growth factor gradients.

## Methods

### Bioinformatics

A total of 47,232 small RNA clone sequences distributed over 65 tissues, including the kidney glomerulus, were obtained from a recent survey [16]. Two compendia with microarray data from mouse tissues, including lung [17,18], were downloaded from the NCBI Gene Expression Omnibus repository. To ensure consistent mapping between datasets, clone/probe sequences were re-annotated against miRBase release 10.1 [19] using a proprietary Matlab (Mathworks Inc. Natick, MA, USA) script. For each mature miRNA, a *P*-value for over-representation in the glomerulus library compared to the other tissues was calculated using Fisher's exact test. Likewise, *P*-values for differential expression in the lung compared to remaining adult tissues were determined using the Student's *t*-test. The *t*-test provides a useful metric of differential tissue expression, although the formal requirements for the underlying distribution of the data may not be completely met [20]. Genomic localization of miRNAs was evaluated using data derived from the UCSC browser (July 2007 assembly) [21].

### Isolation of CD31+ microvascular fragments and TaqMan qRT-PCR

Microvascular fragments were isolated from mouse tissues and embryonic stem cell cultures using mechanical and enzymatic digestion followed by incubation with magnetic Dynabeads coated with anti-CD31 (anti-platelet endothelial cell adhesion molecule (PECAM)). The procedure was performed essentially as described previously [22]. All mice were adult (8 to 12 weeks old) males, either wild-type C57BL/6 or *Pdgfb*^*ret*/*ret *^backcrossed for seven generations onto a C57BL/6 background [23]. RNA from vascular fragments and remaining tissue was prepared using miRNeasy Mini spin columns (Qiagen, Hilden, Germany). Samples were quantified with a NanoDrop spectrophotometer (Thermo Scientific Corporation, Waltham, MA, USA) and cDNA was synthesized using equal amounts of RNA in each reaction (High-Capacity Reverse Transcription Kit or MicroRNA Reverse Transcription Kit, Applied Biosystems, Foster City, CA, USA). Expression levels were determined using pre-designed TaqMan assays (Applied Biosystems) on a 7900 HT real-time PCR system, according to the manufacturer's instructions. Relative levels were calculated using the 2^-Ct ^method. *Fli1 *mRNA levels were determined using SYBR Green quantitative qPCR (95°C, 55°C, 72°C, 40 cycles) using the following primers: 5'-TATCAGATCCTGGGGCCAAC-3' and 5'-CTCATCAGGGTCCGTCATTT-3'.

### Differentiation of embryonic stem cells into vascular sprouts

The murine embryonic stem cell line R1 [24] was routinely cultured on growth arrested mouse embryonic fibroblasts in stem cell medium composed of DMEM-Glutamax (Invitrogen, Carlsbad, CA, USA) supplemented with 25 mM HEPES pH 7.4, 1.2 mM sodium pyruvate, 19 mM monothioglycerol (Sigma-Aldrich, St. Louis, MO, USA), 15% fetal bovine serum (Gibco/Invitrogen, Carlsbad, CA, USA), and 1,000 U/ml leukemia inhibitory factor (Chemicon International/Millipore, Billerica, MA, USA). EBs were generated by aggregation of stem cells in hanging drops in the absence of leukemia inhibitory factor, as described previously [25]. Briefly, EBs were collected after 4 days and seeded into 12-well dishes onto a layer of 0.9 ml solidified collagen type I solution composed of Ham's F12 medium (Promocell, Heidelberg, Germany), 6.26 mM NaOH, 20 mM HEPES, 0.117% NaHCO_3_, 1% Glutamax-I (Gibco) and 1.5 mg/ml collagen I (PureCol, Advanced BioMatrix, San Diego, CA, USA). Immediately thereafter, a second layer of 0.9 ml collagen solution was added on top and allowed to polymerize. After 3 hours, 0.9 ml of stem cell medium supplemented with vascular endothelial growth factor A (VEGFA; PeproTech, Rocky Hill, NJ, USA), at a final concentration of 30 ng/ml, was added to induce angiogenic sprouting. The medium was replaced every second day. EBs were excised from the gels at day 14 and immediately processed for isolation of CD31+ vascular fragments, as described above. NG2+ cells were isolated with the same protocol using a rabbit anti-rat NG2 antibody (Chemicon; AB5320), after depletion of CD31+ cells from the cultures.

### *In situ *hybridization and immunohistochemistry

*In situ *hybridization was performed using a 3' DIG-labeled miRCURY LNA probe to mouse miR-145 and miR-126 (Exiqon, Vedbaek, Denmark) as previously described [26]. For dual detection of miR-145 and the pericyte marker NG2, the immunostaining was performed after development of the *in situ *signal. Slides were washed in phosphate-buffered saline, blocked with 3% donkey serum and 1% bovine serum albumin in phosphate-buffered saline, then incubated with rabbit anti-rat NG2 antibody (Chemicon; diluted 1/50) overnight at 4°C, washed in phosphate-buffered saline, then detected with Alexa488-conjugated donkey anti-rabbit IgG (Invitrogen; diluted 1/200).

### Vascular aortic endothelial cell culture, scratch wound and proliferation assays

Mouse vascular aortic endothelial cells (VAECs; Dominion Pharmakine, Derio-Bizkaia, Spain) were cultured in RPMI 1640 media (Sigma) supplemented with 10% fetal calf serum (Gibco), 1 μg/ml dexamethasone, 10 U/ml heparin, 50 U/ml penicillin/streptomycin and 75 μg/ml EC growth factor supplement (Sigma). For scratch wound migration assays, cells were transfected by electroporation (Nucleofector system, Basic Endothelial Cell Kit, Amaxa Inc/Lonza group ltd, Basel, Switzerland) using 0.5 μg of synthetic mature miR-145 double-stranded RNA (dsRNA; Pre-miR-145; Applied Biosystems) or negative control dsRNA (Stealth siRNA negative control; Applied Biosystems), seeded onto 6-well plates and cultured for 48 hours. Scratch wounds were generated in the cell monolayer using a pipet tip and each wound was photographed at 0 and 24 hours. Wound widths were evaluated blindly at both time-points and the average amount of closure was determined for each replicate transfection. VAEC proliferation was measured by quantification of 5'-bromo-2'-deoxyuridine (BrdU) incorporation. Cells were pulsed for 4 hours with 20 μM BrdU and DNA synthesis was determined using a colorimetric ELISA (Calbiochem/Merck, Darmstadt, Germany) according to the manufacturer's instructions. Absorbance was measured at dual wavelengths of 450 to 540 nm.

### Microfluidic migration chamber

Migration of HUVECs in response to a stable gradient of VEGFA-165 (PeproTech; 0 to 50 ng/ml over a distance of 400 μm) or BJ-hTERT (human foreskin fibroblast) cells in response to platelet-derived growth factor (PDGF)-BB (0-20 ng/ml) was examined using a microfluidic chemotaxis chamber, essentially as previously described [27]. HUVECs were transferred to 3-cm culture dishes coated with type A gelatin from porcine skin (Sigma) and were allowed to attach to the dish in EGM-2MV medium (Lonza) with serum and supplement growth factors. After 2 hours the medium was aspirated and the cells were transfected with 0.5 μg of Pre-miR negative control, Pre-miR-145, Anti-miR negative control or Anti-miR-145 (Applied Biosystems) using siPORT *NeoFX *(Ambion, Austin, TX, USA) in serum and growth factor free EBM-2 medium (Lonza) containing 0.2% bovine serum albumin. After 24 hours the gradient experiment was initiated. BJ-hTERT cells were cultured in minimal essential medium (MEM, Invitrogen) containing 10% fetal calf serum (Gibco), 1 mM sodium pyruvate (Gibco) and non-essential amino acids (Gibco). Cells were transfected using electroporation (0.5 μg, Nucleofector system, Amaxa) and were allowed to rest between 24 and 48 h before being seeded onto gelatin A-coated culture dishes and serum starved overnight, before onset of gradient. VEGFA-165 or the PDGF-BB gradients were generated in serum-free cell medium. Cell migration was tracked during 3 hours (HUVECs) or 4 hours (BJ-hTERT cells) using a Cell Observer System (Carl Zeiss AB, Stockholm, Sweden) fitted with a Zeiss Axiovert 200 microscope, an AxioCam MRm camera, a motorized X/Y stage, and an XL incubator with equipment for temperature and CO_2 _control (Zeiss). Cells were kept in a humidified atmosphere of 5% CO_2 _in air at 37°C during all experiments. AxioVision software (Zeiss) was used for time-lapse imaging and cell tracking.

### Luciferase reporter assays

Oligonucleotides (65 bp) harboring wild-type or mutated miR-145 binding sites from the mouse *Fli1 *3' UTR (Additional data file 1) were annealed and ligated into the *Hin*dIII and *Spe*I sites of the pMIR-REPORT CMV-firefly luciferase reporter vector (Applied Biosystems). All constructs were verified by sequencing. HEK293 cells were seeded onto 24-well plates at a density of 50,000 cells/well and cultured overnight in DMEM (10% fetal calf serum) without antibiotics. Cells were transfected with 60 ng of pMIR-REPORT, 8 ng of pRL-SV40 renilla luciferase control vector and 10 pmol of Pre-miR negative control or Pre-miR-145 using Lipofectamine 2000 (Invitrogen) and luciferase activity was assayed after 48 hours using the Dual-Luciferase Reporter System (Promega, Madison, WI, USA).

Long mouse and human *Fli1 *3' UTR fragments were amplified by PCR using the following primers (numbers indicate the position starting from the stop codon): 5'-AACTAACACCAGTTGGCCTTC-3' and 5'-CGTCAGGAGTGTCTGAGTTTG-3' (1-704); 5'-GCTTCTTCTAGCTGAAGCCCATC-3' and 5'-GTCAAATTATTTTACAACATGG-3' (3-1,391). Amplimers were cloned in psiCHECK-2 (Promega) to generate *Renilla *luciferase-3' UTR reporter constructs. Basal expression of firefly luciferase from the same plasmid served as an internal control. HEK293T cells seeded in 96-well plates were cotransfected with plasmid (50 ng per well) and synthetic miRNA (0.25 to 2.5 pmol per well; Biomers, Ulm, Germany) using Lipofectamine 2000. Luciferase activity was assayed 24 hours after transfection as described [28]. Nucleotides 2 and 4 in the seed region of three predicted miR-145 sites within the mouse 3' UTR fragment were mutated using Multisite-Quickchange (Stratagene/Agilent, Santa Clara, CA, USA). Results represent *Renilla*/firefly luciferase ratios from four independent experiments performed in triplicates. Statistical significance was evaluated using Student's *t*-test.

### Western blot analysis

VAECs were electroporated with either Pre-miR-145 or Pre-miR negative control as described above. Nuclear extracts were prepared using the CelLytic NuCLEAR kit (Sigma) at 72 hours post-transfection. Western blotting was performed using a Fli1 antibody (Sc-356; Santa Cruz Biotechnology, Santa Cruz, CA, USA) at 2 mg/ml and ECL reagents (Amersham Biosciences/GE Healthcare Bio-Sciences, Uppsala, Sweden). As a loading control, the membrane was stripped and reprobed using a lamin A/C antibody (Sc-7293, Santa Cruz Biotechnology) at a dilution of 1/1,500. Densitometric analysis was performed using ImageJ software.

## Results

### Bioinformatic prediction of microvascular miRNAs

**Figure 1:**
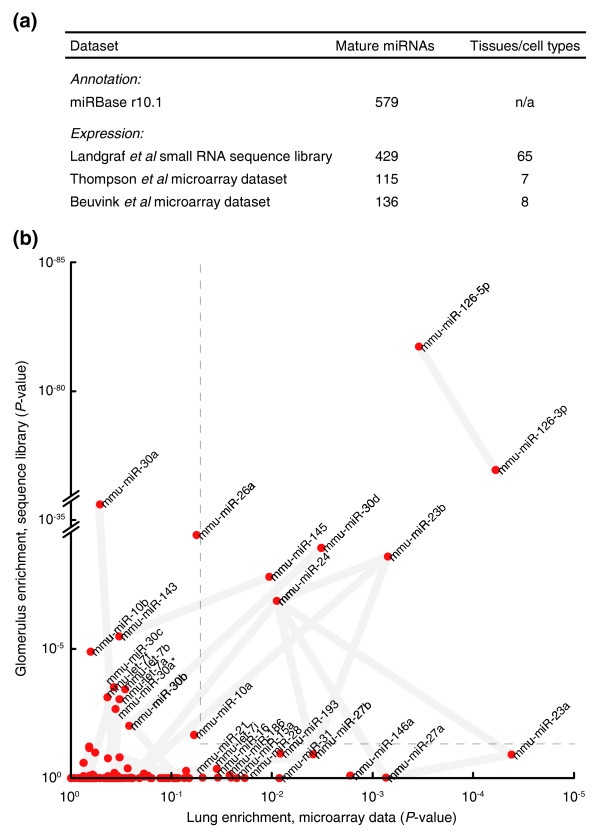
Identification of putative microvessel-enriched miRNAs using public expression data. **(a) **Table of datasets included in the analysis. Mature miRNAs were evaluated for enrichment in the lung in two datasets (Thomson *et al. *[17] and Beuvink *et al. *[18]). Glomerular enrichment was determined in an expression dataset derived by small RNA library sequencing (Landgraf *et al. *[16]). All clone and probe sequences were re-annotated against the miRBase microRNA repository [19]. **(b) **Scatter plot showing mature miRNAs enriched in both the glomerulus (y-axis) and lung (x-axis; using best value from the two microarray datasets). miRNAs connected by thick grey lines are co-localized in the genome (<10 kb) and likely to be co-transcribed.

Protein-coding genes with selective expression in the microvasculature were identified in a recent study based on their enrichment in the lung and in the kidney glomerulus [29]. Differential expression in both of these endothelium-rich tissues minimized contamination by epithelial transcripts and permitted identification of numerous known and novel microvascular markers. Here we applied a similar strategy to identify candidate microvascular-enriched miRNAs. Data were gathered from three different sources: a set of small RNA sequence libraries of varying sizes covering 65 mouse tissues, including the glomerulus [16], and two compendia with microarray data from adult mouse tissues, including lung [17,18] (Figure 1a). miRNAs were scored for enrichment in glomerulus and lung and this formed the basis of our selection (Additional data file 2).

Among those with favorable scores in this analysis, miR-126-3p and miR-126-5p (the two mature forms of miR-126) stood out as strongly enriched in both glomerulus and lung (Figure 1b). Several other miRNAs also appeared as promising candidates for selective vascular expression, including miR-145, miR-30d, miR-23b and miR-24 (within the dashed lines in Figure 1b). miRNAs connected by thick grey lines in the figure are co-localized in the genome (<10 kb) and likely derive from the same polycistronic transcript [30].

### Differential expression of miR-126, miR-145, miR-24, and miR-23a in the mature microvasculature

**Figure 2:**
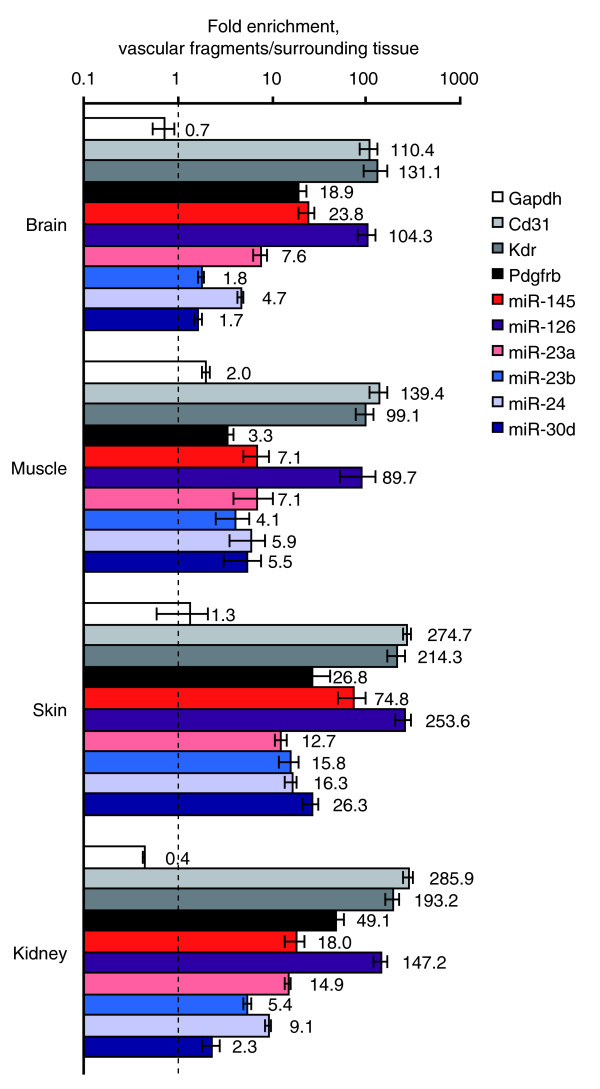
Differential expression of miRNAs in CD31+ vascular fragments isolated from mature mouse organs. Anti-CD31-coated magnetic beads were used to isolate microvascular fragments from adult (8 weeks) C57Bl/6 mouse organs. cDNA was prepared from the fragments and the remaining tissue using equal amounts of RNA, and miR-145 expression levels were determined using TaqMan qRT-PCR. The figure shows average paired expression ratios between fragments and surrounding tissue ± standard error of the mean (n = 4, 3, 2 and 4 for brain, muscle, skin and kidney, respectively). GAPDH, CD31 and VEGFR-2 (Flk1) TaqMan assays were used as quality controls.

Based on the above described *in silico *analyses, we chose to further characterize the expression of miR-126-3p (the predominant mature form of this miRNA, hereafter referred to as miR-126), miR-145, miR-30d, miR-23b, miR-24 and miR-23a; the latter being co-transcribed with miR-24 [1]. Microvascular fragments were isolated from adult mouse tissues using mechanical and enzymatic digestion followed by separation using anti-CD31 (PECAM)-coated magnetic beads. RNA was prepared from the fragments and the remaining tissue fractions. qRT-PCR analysis showed that miR-126 was highly differentially expressed in CD31+ fragments in all adult organs assayed, with fragment-to-surrounding tissue ratios ranging from 90 to 250. These ratios are in parity with the endothelial markers Cd31 and Kdr (Vegfr2/Flk1; Figure 2). The remaining miRNAs were also enriched in vascular fragments to varying degrees. In particular, miR-145 showed consistent and high differential expression in microvessels (24-, 7-, 75- and 18-fold for brain, muscle, skin and kidney, respectively). In addition, miR-23a and miR-24 were consistently differentially expressed, with enrichments ranging from 5- to 16-fold. Gapdh, included as a control, showed weak or no enrichment across the panel.

### miRNA expression during vascular formation

**Figure 3:**
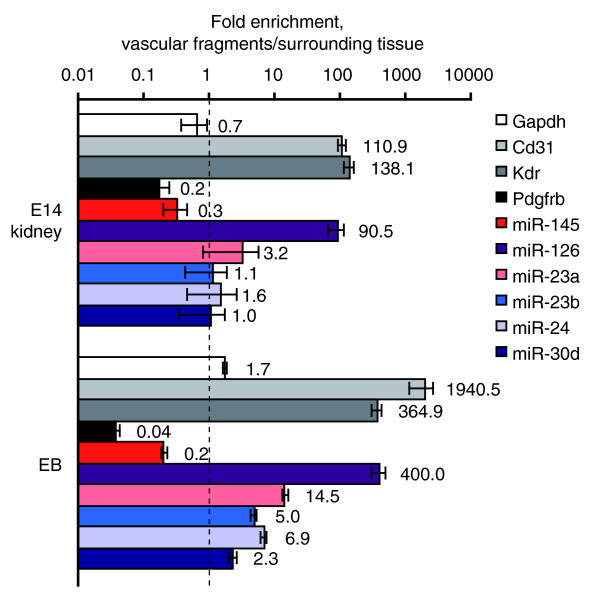
miRNA expression in immature blood vessels. To investigate the expression in immature vessels, microvascular fragments were isolated with anti-CD31-coated magnetic beads from embryonic kidney (E14 kidney) and EBs with active sprouting angiogenesis (n = 3 for both kidney and EB; error bars indicate standard error of the mean).

To evaluate miRNA expression in immature blood vessels, CD31+ microvascular fragments were isolated from mouse kidneys at embryonic day 14, as well as from VEGFA-induced angiogenic sprouts formed in EB cultures. miR-126 showed strong enrichment in CD31+ fractions from both tissues (Figure 3). miR-23a and miR-24 were enriched in sprouts from EBs but not in fragments from embryonic day 14 kidneys. miR-145, in contrast, was predominantly expressed in the leftover fractions. The pericyte marker Pdgfrb showed a similar pattern with strong enrichment in CD31+ fragments from adult tissues but not in embryonic vascular fragments (Figures 2 and 3), which suggests that miR-145 could be expressed by pericytes.

### miR-145 is selectively expressed by pericytes

**Figure 4:**
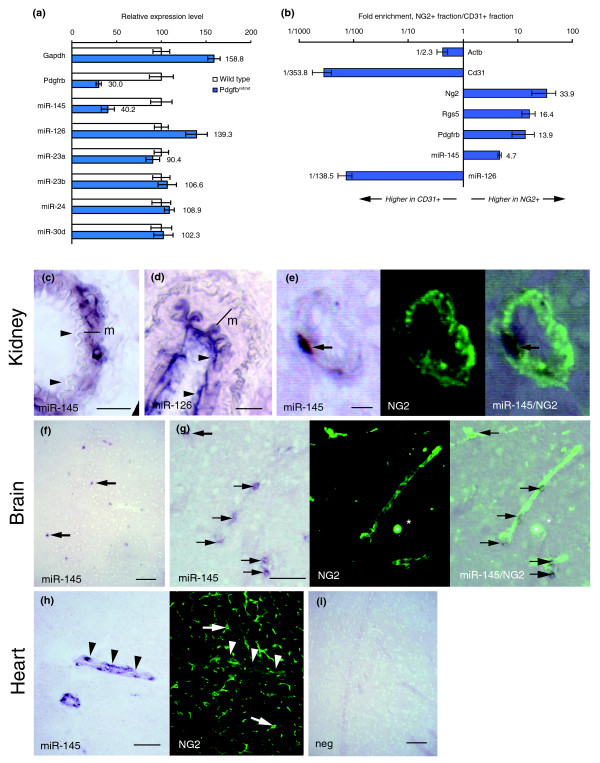
Pericyte expression of miR-145. **(a) **To differentiate between pericyte and EC expression, vascular fragments were isolated from the brains of pericyte-deficient *Pdgfb*^*ret*/*ret *^mice using anti-CD31-coated magnetic beads. Bars show relative expression levels in CD31+ fragments from wild-type and *Pdgfb*^*ret*/*ret *^± standard error of the mean (n = 4 and 3, respectively). **(b) **CD31+ cells were isolated from EB cultures using magnetic beads. After depletion of CD31+ cells, cells expressing the pericyte marker NG2 were isolated using the same protocol. Bars show the ratio of expression between NG2+ and CD31+ fragments. Error bars indicate standard error of the mean (n = 3). **(c-i) ***In situ *hybridization (blue) against miR-145 (c, e-h) and miR-126 (d) with double staining for NG2 (green) (e, g-h). (c) miR-145 *in situ *hybridization stains vascular smooth muscle cells (m, media) whereas (d) miR-126 stains ECs (arrowheads) in kidney artery (scale bar, 25 μm). (e) High power magnification of a small vessel in kidney shows that a miR-145-positive cell (arrow) expresses NG2 (scale bar, 5 μm). (f) miR-145 *in situ *hybridization labels solitary cells in adult brain (arrows; scale bar, 100 μm). (g) Double staining for miR-145 (arrows) and NG2 show co-expression in cells tightly associated with small caliber (10 μm) capillaries in brain (scale bar, 50 μm). (h) miR-145 staining in the heart is confined to arterioles (arrowheads) whereas no expression was detected in NG2-positive cells in microvessels (arrows) (scale bar, 50 μm). (i) Negative control (without probe; scale bar, 100 μm).

To test the hypothesis that miR-145 is expressed by pericytes, CD31+ fragments were purified from the brains of Pdgfb retention-motif mutant mice (*Pdgfb*^*ret*/*ret*^) that lack a stretch of basic amino acids in the carboxyl terminus of PDGF-B. These mice display defective pericyte investment of microvessels [23]. As expected, *Pdgfrb *mRNA levels were reduced in *Pdgfb*^*ret*/*ret *^vascular fragments compared to wild-type mice (*P *= 0.001; Figure 4a). Expression of miR-145 was also reduced in mutant microvessels (*P *= 0.008), whereas no notable differences were observed for the other miRNAs. These results gave further support to the idea that microvascular miR-145 expression is derived primarily from pericytes.

As a complementary approach, CD31+ ECs and NG2+ pericytes were isolated from EB cultures. Expression levels were determined using qRT-PCR and the ratio of the signals from the two fractions was determined. In accordance with the pericyte markers, miR-145 expression was higher in NG2+ cells compared to CD31+ cells (Figure 4b). In contrast, the endothelial marker Cd31 and miR-126 were highly enriched in the CD31+ fraction.

Next, we performed *in situ *hybridization on tissue sections using probes specific to miR-145 and miR-126. As expected, miR-145 stained smooth muscle cells in larger vessels whereas miR-126 stained ECs (staining patterns in kidney arteries are shown in Figure 4c, d). In brain parenchyma, miR-145 showed staining in solitary scattered cells, consistent with expression in pericytes (Figure 4f). Double staining using an NG2 antibody confirmed co-expression of the two molecules in brain capillaries (Figure 4g) and in small caliber blood vessels in the kidney (Figure 4e). There was, however, no detectable expression of miR-145 in pericytes in the heart, where the expression was confined to arterioles and larger vessels (Figure 4h). Compared to miR-145, NG2 staining indicated larger areas in kidney and brain microvessels (Figure 4e, g). This suggests that miR-145 is expressed by a subset of pericytes. However, this could also be explained by NG2's subcellular distribution in pericyte processes that extend from the main cell body and cover the capillary cell surface.

We conclude that miR-145 is selectively expressed in microvessel pericytes whereas the remaining miRNAs are expressed in ECs.

### *Fli1 *is a target of miR-145

**Figure 5:**
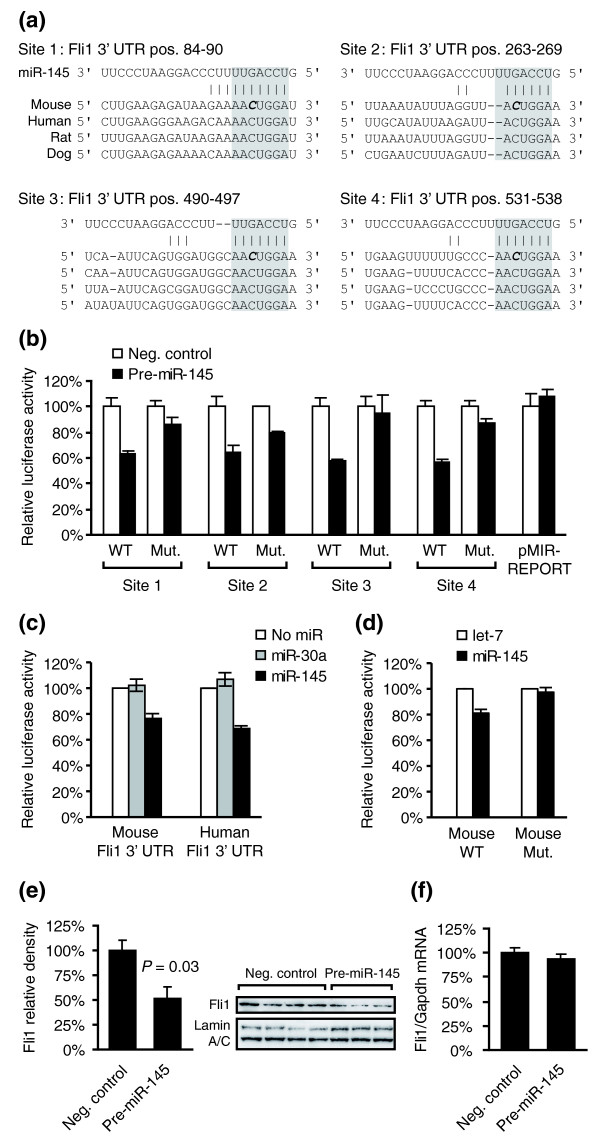
Regulation of Fli1 by miR-145. **(a) **Four possible miR-145 binding sites were identified in the *Fli1 *3' UTR. Evolutionary conservation across four mammalian species is shown. Seed regions are indicated by grey boxes. **(b) **Luciferase assays show that the predicted sites can mediate silencing by miR-145. Approximately 60-bp regions containing wild-type (WT) miR-145 binding sites in the *Fli1 *3' UTR were cloned into pMIR-REPORT vector (Applied Biosystems). Identical constructs with single base-pair mutations (Mut) were generated (mutated bases, C to G, are indicated in italics and bold in the sequences). HEK293 cells were co-transfected with pMIR-REPORT and either negative control dsRNA or a synthetic miR-145 dsRNA (Pre-miR-145) and luciferase activity assayed after 48 hours. Signals were normalized to the control groups. Error bars indicate standard error of the mean (n = 2, *P *< 0.05 for control versus Pre-miR-145 with all WT constructs). **(c) **Larger regions of the mouse and human *Fli1 *3' UTRs (704 and 1,288 bp, respectively) were cloned into luciferase reporter vectors and luciferase activity was assayed 24 hours after cotransfection with synthetic miR-30a or miR-145 in HEK293 cells. Four replicate experiments were performed and values shown are normalized to the empty (plasmid only) transfections (*P *< 0.001 for both constructs, comparing empty and miR-145 transfections). **(d) **Site-directed mutagenesis was applied to the 704-bp mouse *Fli1 *3' UTR fragment. Two single base mutations were introduced in each of the seed regions of predicted target sites 2 to 4. Constructs were cotransfected with either synthetic let-7f (control) or miR-145 and luciferase activity was assayed after 24 hours (n = 4). **(e) **Relative Fli1 protein levels in VAECs were measured 72 hours post-transfection with either Pre-miR-145 or a dsRNA control. Nuclear extracts were prepared and expression was assayed by western blotting followed by densitometric analysis. The membrane was re-probed with a lamin A/C antibody as a loading control. Error bars indicate standard error of the mean. **(f) ***Fli1 *mRNA levels in VAECs 72 hours post-transfection were determined using qRT-PCR and normalized to GAPDH. Error bars represent standard error of the mean (n = 4).

miRNA target prediction software was used to identify possible targets for miR-145. The highest-scoring predicted target using the miRanda algorithm [31] was the gene encoding the Ets transcription factor Friend leukemia integration 1 (*Fli1*). *Fli1 *also scored favorably using picTar [32] and TargetScan [33], the latter identifying four evolutionarily conserved miR-145 binding sites in the *Fli1 *3' UTR (Figure 5a).

To evaluate if the predicated sites can bind miR-145 and induce silencing, we generated a series of eight constructs, each consisting of a CMV-luciferase reporter followed by a portion of the *Fli1 *3' UTR containing a wild-type or mutated site. Transfection of a synthetic miR-145 mimic dsRNA (Pre-miR-145) into HEK293 cells significantly reduced reporter activity for all predicted sites (Figure 5b). Single base-pair mutations reduced or abolished the effect of miR-145 on reporter activity in all cases. An empty reporter vector, lacking a cloned target site in the 3' UTR, was not affected by miR-145 overexpression.

Constructs with either a full length human or a long (700 bp) fragment of the mouse *Fli1 *3' UTR were generated to evaluate the predicted sites in their natural sequence context. Cotransfection with a miR-145 mimic significantly reduced reporter activity compared to transfection without a miRNA or with an unrelated miRNA (Figure 5c). The effect was abolished when mutations were introduced in three out of four predicted sites in the mouse 3' UTR (Figure 5d).

An effect of miR-145 on endogenous Fli1 protein levels was demonstrated in VAECs. Western blot analysis 72 hours post-transfection of Pre-miR-145 showed that Fli1 protein levels were decreased compared to cells treated with Pre-miR negative control (Figure 5e). Since miRNAs can induce both translational repression and target mRNA degradation, we performed qRT-PCR to assess the expression of *Fli1 *mRNA after introduction of Pre-miR-145 or Pre-miR negative control. No significant reduction was observed (Figure 5f). Translational repression without mRNA degradation has been described for numerous miRNAs, and our findings are consistent with a previous report suggesting that miR-145 is primarily a repressor of translation [34]. VAECs were also transfected with Anti-miR-145 in a loss-of-function experiment. This did not affect Fli1 levels (data not shown), which is consistent with low endogenous expression of miR-145 in this cell type (Additional data file 3).

### miR-145 modulates cell migration in vitro

**Figure 6:**
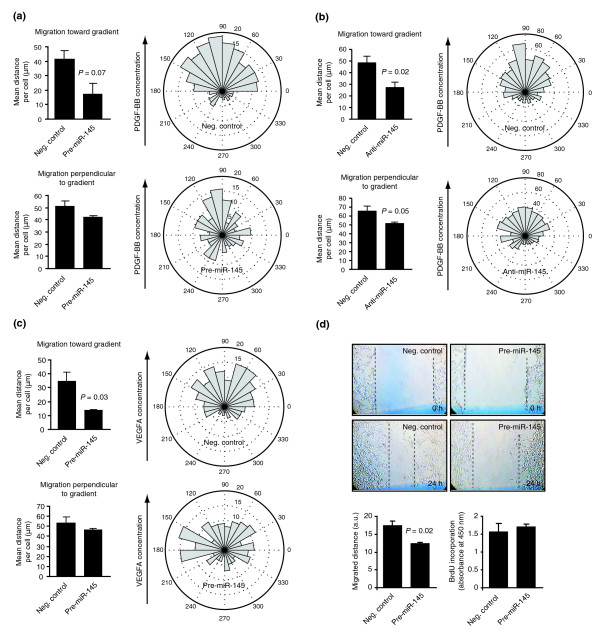
Elevated levels of miR-145 leads to reduced microvasular cell migration. **(a) **Migration of BJ-hTERT cells was evaluated using a microfluidic chemotaxis chamber. Individual cells, cultured in a stable PDGF-BB gradient (0 to 20 ng/ml over a distance of 400 μm), were tracked using time-lapse microscopy. Cells were transfected with control dsRNA or Pre-miR-145 and average migrated distances toward the gradient and perpendicular to the gradient were calculated. The bar graphs show average values from three independent experiments ± standard error of the mean (*P*-value obtained using the two-tail *t*-test). The polar plots illustrate the direction of migration for individual cells in the control experiments (top) and in the Pre-miR-145 transfected cultures (bottom). The radius of each 15 degree sector indicates the number of cells that migrated in this direction. A total of 285 and 239 cells were tracked for the negative control and Pre-miR-145, respectively. **(b) **Migration of Bj-hTERT cells transfected with control single-stranded RNA or Anti-miR-145 in a PDGF-BB gradient, as described above for Pre-miR-145. The bar graphs show average results from five independent experiments, and a total of 701 and 622 cells were tracked for the negative control and Anti-miR-145, respectively. **(c) **Migration of HUVECs in response to a VEGFA-165 gradient (0 to 50 ng/ml), as described above for PDGF-BB. Results are average values from three independent experiments, and a total of 185 and 191 cells were tracked for the negative control and Pre-miR-145, respectively. **(d) **Migration of VAECs was evaluated using scratch wound assays. Cells were electroporated with either a negative control dsRNA or a synthetic miR-145 dsRNA (Pre-miR-145) and cultured for 48 hours. A scratch wound was generated in the cell monolayer and the degree of wound closure determined 24 hours later. The graph shows the mean migrated distance (difference in wound width after 24 hours ± standard error of the mean, n = 3). Proliferative activity of VAECs 48 hours post-transfection was assessed by quantification of BrdU incorporation. Cells were pulsed for 4 hours and incorporated BrdU was measured using a colorimetric ELISA (mean absorbance ± standard error of the mean; n = 4).

In order to assess the role of miR-145, functional assays were performed in human foreskin fibroblasts, and in ECs that express Fli1. Cell migration is often guided by growth factor gradients *in vivo*. PDGF-BB is known to stimulate migration of several different cell types, including smooth muscle and fibroblasts [35,36]. It is also a key regulator of pericytes *in vivo *[37]. We therefore investigated cell migration in response to a stable gradient of PDGF-BB using a microfluidic chemotaxis chamber. Human foreskin fibroblasts (BJ-hTERT) were transfected with Pre-miR-145 or Pre-miR negative control in gain-of-function experiments and with Anti-miR-145 or Anti-miR-control in loss-of-function experiments (expression levels of miR-145 in BJ-hTERT cells are presented in Additional data file 3). Individual cells were tracked using time-lapse microscopy during 3 hours [27]. The average migrated distance per cell toward the high-end of the PDGF-BB gradient was reduced by more than 50% in Pre-miR-145 transfected cells, whereas migration perpendicular to the gradient was only slightly, and not significantly, reduced (Figure 6a). Similarly, migration towards the high-end of the gradient was reduced by Anti-miR-145 (Figure 6b). Migration perpendicular to the gradient was also significantly reduced by this treatment.

To investigate the effect of miR-145 on VEGFA-165-induced migration, HUVECs were cultured in a stable gradient of VEGFA-165. Control cells migrated consistently toward the high-end of the gradient, whereas Pre-miR-145-transfected cells exhibited a clear (>50%) reduction in migration in this direction (Figure 6c). Migration perpendicular to the gradient was not significantly reduced.

Migration was also evaluated on VAECs cultured in EC growth factor supplemented medium using a wound healing assay. Migration was reduced in Pre-miR-145 transfected cells compared to cells transfected with a dsRNA control (Stealth siRNA control, Invitrogen; Figure 6d). However, proliferation rate, as determined using a BrdU ELISA assay, was not affected. These findings point to a role for miR-145 in regulation of cell migration.

## Discussion

By screening for mature miRNAs with vascular expression patterns we found that miR-145, miR-126, miR-23a, and miR-24 were enriched in the microvasculature *in vivo*. miR-145 was specifically expressed in pericytes, whereas the others were expressed in ECs. We demonstrated that the Ets factor Fli1 is a regulatory target of miR-145 and that perturbed levels of miR-145 reduced cell migration. The present study provides insight into microvascular-selective miRNA expression and differs from previous screens due to its *in vivo *focus.

There is a notable overlap between high-scoring miRNAs in our screen and those identified by several *in vitro *microarray studies of HUVECs. Many of the miRNAs we identified scored favorably in one or more of these screens, including miR-23a [6-8,12], miR-23b [7,8,12], miR-24 [7,8,12] and miR-126 [6,8,12]. In addition, miR-23a, miR-23b, miR-24 and miR-30d were shown to be upregulated in hypoxia [38]. miR-126, for which an important functional role in the endothelium has already been firmly established [8-11], stood out as strongly enriched in microvascular fragments from mature mouse tissues as well as in tissues undergoing active angiogenesis.

miR-145 has previously been shown to be selectively expressed in smooth muscle cells [39-41]. It controls phenotypic modulation of these cells by inducing expression of contractile proteins, an effect that is partly mediated by targeting Klf5 [39,41]. Forced expression of pre-miR-145 also reduced neointimal formation after arterial injury [41]. Here, we show that miR-145 is expressed in microvascular pericytes. miR-145 was expressed in scattered NG2-positive cells tightly associated with the smallest caliber capillaries in the brain and kidney. This staining pattern is typical for pericytes and not compatible with vascular smooth muscle cells. Furthermore, expression of miR-145 was reduced in vascular fragments isolated from Pdgfb^ret/ret ^mice and enriched in NG2+ cells isolated from embryoid bodies. We did not, however, detect miR-145 in pericytes in the heart, where expression was confined to larger arterioles.

Perturbed expression of miR-145 reduced cell migration in cultured fibroblasts and ECs. This finding is supported by a recent publication that describes miR-145 knockout mice [40]. These mice show reduced neo-intima formation in response to ligation of the carotid artery. The authors suggest that the phenotype is caused by failed migration of medial smooth muscle cells, although no migration experiments were performed. In the same publication miR-145 is shown to selectively target genes that regulate the actin cytoskeleton, which is intimately coupled to cell migration. Several target genes that regulate actin polymerization or depolymerization were identified, some of which have been shown to inhibit migration (*Srgap1 *and *Srgap2*) and others that stimulate migration (*Add3 *and *Ssh2*). Many additional target genes that affect actin dynamics were predicted, which further supports a role for miR-145 in regulation of cell motility. Paradoxically, migration was reduced by both over-expression and silencing of miR-145 in our experiments. The primary role of miR-145 may be to maintain cytoskeleton homeostasis, and perturbed expression levels of miR-145, in either direction, may disturb this balance and negatively affect the cells' ability to remodel the actin cytoskeleton. In zebrafish, loss or gain-of-function experiments with miR-145 leads to identical phenotypes with poorly developed smooth muscle cells in the gut [42].

Considering that miR-145 is selectively expressed by pericytes, it is intriguing that the endothelial and hematopoietic transcription factor Fli1 was identified as a target of miR-145. Fli1 is an early marker of hemangioblast differentiation and plays an important role in blood/vascular development and angiogenesis [43-46]. Recent studies show that hematopoietic cells can emigrate from the circulation and differentiate to pericytes [47-51]. It is tempting to speculate that miR-145 could make such transitions sharp and distinct by silencing the hematopoietic differentiation factor Fli1.

miRNA-based therapeutics is showing promise in animal models and elevation or inhibition of miR-126 has been proposed as a possible therapeutic strategy in ischemic heart disease, cancer, retinopathy and stroke [9]. The miRNAs identified in the present study - miR-145, miR-30D, miR-24, miR-23a and miR-23b - are therefore possible targets in future therapeutic strategies.

## Conclusions

We identified miR-145, miR-126, miR-24 and miR-23a as enriched in microvessels, and showed that microvascular expression of miR-145 is due to its presence in pericytes. We also performed a functional characterization of miR-145 and could show that it is a regulator of Fli1, and that increased or decreased expression of miR-145 leads to reduced cell migration in response to growth factor gradients.

## Abbreviations

BrdU: 5'-bromo-2'-deoxyuridine; dsRNA: double-stranded RNA; EB: embryoid body; EC: endothelial cell; HUVEC: human umbilical vein endothelial cell; miRNA: microRNA; PDGF: platelet-derived growth factor; qRT-PCR: real-time quantitative reverse transcription PCR; UTR: untranslated region; VAEC: mouse vascular aortic endothelial cell; VEGFA: vascular endothelial growth factor A.

## Competing interests

The authors declare that they have no competing interests.

## Authors' contributions

EL designed and performed research, performed bioinformatical analyses, participated in vascular fragment isolation from tissues, performed qRT-PCR analyses on fragments, performed scratch wound and proliferation assays, built reporter constructs and performed reporter assays, performed immunoblot analysis, collected and analyzed data and drafted the manuscript; PFF designed and performed research, isolated vascular fragments, performed miRNA expression analysis, and together with IB planned and performed microfluidic cell migration assays; JH designed and performed research, isolated vascular fragments, and performed qRT-PCR analysis on BJ-hTERT cells; CB performed vascular fragment isolation from tissues; GG isolated vascular fragments from *Pdgfb *mutant mice; CA performed *in situ *hybridizations; PG planned and analyzed experiments with fibroblasts; CK, BS and TB built reporter constructs and performed reporter assays; SJH performed *in situ *hybridizations and edited the manuscript; JK and PL designed and performed research, supervised the project, provided research funding and edited the manuscript. All authors read and approved the final manuscript.

## Additional data files

The following additional data are available with the online version of this paper: a table listing sequence information on predicted miR-145 target sites in the mouse *Fli1 *3' UTR (Additional data file 1); a table listing the statistics on enrichment of miRNA in glomerulus and lungs (Additional data file 2); a figure showing expression levels of miR-145 in BJ-hTERT cells after transfection with miR-145 mimic or inhibitor, and expression levels of miR-145 and Fli1 in BJ-hTERT cells and endothelial cells, respectively (Additional data file 3).

## Supplementary Material

Additional data file 1 Sequence information on predicted miR-145 target sites in the mouse *Fli1 *3' UTR. Click here for file

Additional data file 2 Statistics on enrichment of miRNA in glomerulus and lungs. Click here for file

Additional data file 3 Expression levels of miR-145 in BJ-hTERT cells after transfection with miR-145 mimic or inhibitor, and of miR-145 and Fli1 in BJ-hTERT cells and endothelial cells, respectively. Click here for file
