# Supplementary material for: Discovery of microvascular miRNAs using public gene expression data: miR-145 is expressed in pericytes and is a regulator of Fli1
Source: Genome Med. 2009 Nov 16;1(11):108. doi: 10.1186/gm108 (PMC2808743; doi:10.1186/gm108)
Supplement: Additional data file 3 — Expression levels of miR-145 in BJ-hTERT cells after transfection with miR-145 mimic or inhibitor, and of miR-145 and Fli1 in BJ-hTERT cells and endothelial cells, respectively. [file gm108-S3.PDF]

A

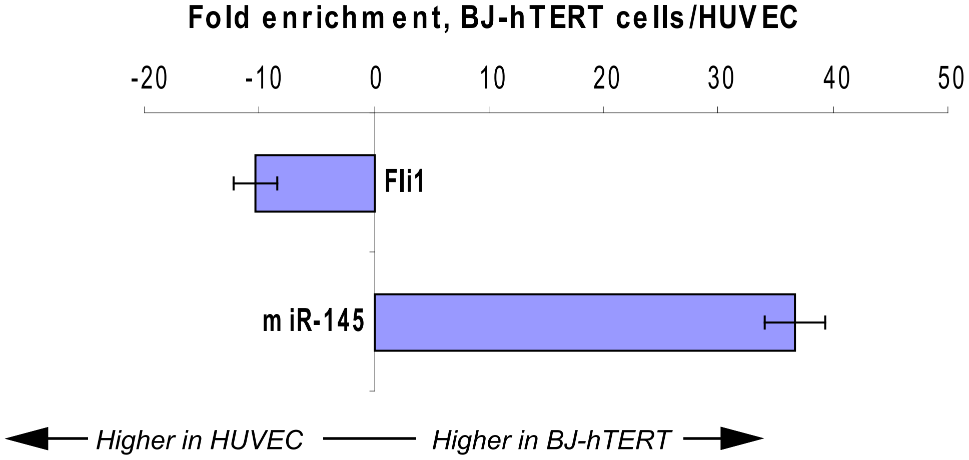

Figure legend: qPCR quantification of endogenous Fli1 mRNA and miR-145 in BJ-TERT cells and HUVEC. Values are presented as mean fold enrichment in BJ-hTERT cells compared to HUVEC. Expression levels were normalized against 18s RNA. Errorbars represent S.E.M. (n=2)

B

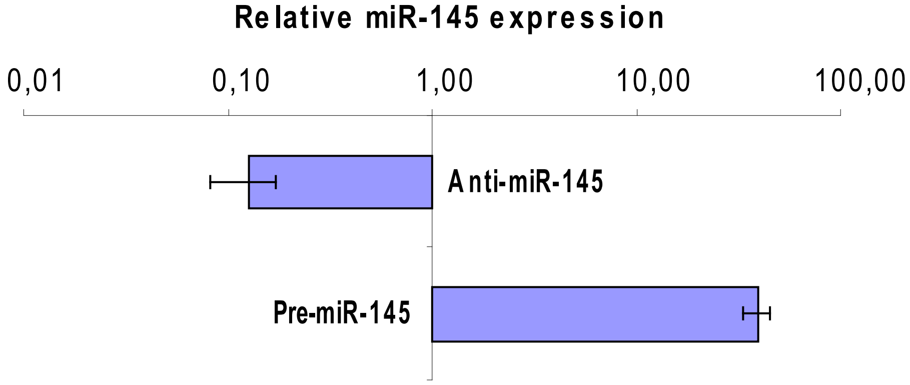

Figure legend: qPCR quantification of miR-145 levels in BJ-hTERT cells after transfection with miR-145 mimic and inhibitor, respectively. Values are presented as mean relative expression (log scale). Errorbars represent S.E.M. (n=3)
